# PROTOCOL: The use of information and communications technologies (ICT) for contributing to the prevention of, and response to, sexual and gender‐based violence against women and children in lower‐ and middle‐income countries: an evidence and gap map

**DOI:** 10.1002/cl2.1153

**Published:** 2021-03-15

**Authors:** William C. Philbrick, Jacob R. Milnor, Madhu Deshmukh, Patricia N. Mechael

**Affiliations:** ^1^ Maestral International Atlanta Georgia USA; ^2^ Evandro Chagas National Institute of Disease Oswaldo Cruz Foundation Rio de Janeiro Brazil; ^3^ CARE Atlanta Georgia USA; ^4^ HealthEnabled Washington District of Columbia USA

## Abstract

This is the protocol for the development of a Campbell Collaboration evidence and gaps map (EGM). The primary objective of this evidence and gap map (EGM) is to answer the following question: (1) What is the evidence connected with the use of information and communications technologies (ICT) for preventing and responding to sexual and gender‐based violence (SGBV) against women and children in lower‐ and middle‐income countries (LMIC)?

(a) the EGM will provide a structured and accessible contextual framework for research to stakeholders and policymakers in SGBV and ICT; (b) the EGM will identify gaps in the available ICT and SGBV evidence; (c) the EGM will identify clusters of evidence suitable for systematic review; and (d) the EGM will look for and build connections between related areas of research in ICT and SGBV.

As part of identifying the evidence connected with the use of ICT for preventing and responding to SGBV we seek to answer the following questions based upon the available evidence:

(a) Does the use of ICT prevent SGBV against women and children in LMIC?
(b) How effective is ICT at improving access to quality services for SGBV survivors in LMIC?
(c) Does the use of ICT contribute to effectively achieving intermediate outcomes that lead to the prevention of SGBV against women and children, and/or improving access for SGBV survivors to response services in LMIC?
(d) What are the enabling factors associated with the implementation of ICT and SGBV interventions?

## 1 BACKGROUND

### 1.1 Introduction

#### 1.1.1 The problem, condition or issue

##### Global sexual and gender‐based violence

The problem of sexual and gender‐based violence (SGBV)^1^ against women and children (regardless of gender) is widespread globally, and particularly prevalent in lower‐ and middle‐income countries (LMIC) where stresses from socio‐economic and political pressures tend to be more exacerbated. Further, prevalence of violence against all women (whether in the form of intimate partner violence [IPV], or perpetrated by non‐partners), tends to be higher in LMIC (World Health Organisation, 2013). Despite higher prevalence, there is relatively less investment in SGBV research in LMIC compared to higher income countries. (Coll et al., 2020).

SGBV is a ubiquitous global phenomenon. It occurs in many forms and contexts including IPV (physical, sexual and emotional abuse); sexual violence; conflict‐related sexual violence; forced and early marriage; trafficking; female genital mutilation; femicide; homophobia and transphobia; and sexual harassment and abuse. What unites these diverse manifestations of violence is that, at their core, they are violent acts associated with the socially assigned gender differences between males and females, often exploiting individual and social power imbalances. SGBV is also often a reaction to perceived threats to the same socially constructed gender roles or norms.

MEASURE Evaluation,^2^ which maintains a key global database on SGBV, states “international studies and survey data confirm that SGBV is a widespread problem with serious repercussions in terms of personal suffering, health complications, disability, and death for women, children and men, in addition to having significant costs for healthcare systems and society at large” (USAID & PEPFAR, 2020). Trauma resulting from SGBV constitutes one of the most profound, but often immeasurable consequences. According to a 2013 study, women who have experienced physical and/or sexual IPV “report higher rates of depression, having an abortion, acquiring HIV, and alcohol use, compared to women who have not” (World Health Organisation, 2013).^3^

Few experts challenge the assessments identifying the consequences of SGBV. Some studies and a systematic review outlining effective interventions and approaches to reducing IPV, for example, have added to the SGBV evidence base. (Michau et al., 2015). Yet, the field is characterised by “gaps in research and evidence” which include “lack of data of violence from certain regions; an incomplete understanding of the full scope of health and other consequences; a limited knowledge of what works to prevent and respond to violence against women and girls; and a general bias of published literature towards high‐income countries” (Temmerman, 2015).

Having complete accurate and timely data on the SGBV is still more of a goal than a reality. Available country data indicates that between 15% and 76% of women are targeted for physical and/or sexual violence in their lifetime (World Health Organisation, 2013). Globally, up to 50% of sexual assaults are committed against girls under 16 (World Health Organisation, 2013). Global data estimates that in 2002 alone, 150 million girls under the age of 18 suffered some form of sexual violence (UN Women, 2019). Femicide, or the intentional killing of women based on their gender, has increased globally since 2012 by roughly 80% (United Nations Office on Drugs and Crime, 2019); with Africa and Latin America having the highest rates per 100,000 female inhabitants (3.1 and 1.6, respectively) (United Nations Office on Drugs and Crime, 2019). Individual countries, such as El Salvador (6.8 per 100 k females) and Honduras (5.1 per 100 k females), have even higher rates (United Nations Gender Equality Observatory for Latin America and the Caribbean, 2020).

For example, countries such as Afghanistan, the Central Africa Republic, Colombia, the Democratic Republic of Congo, Iraq, Libya, Mali, Somalia and Yemen, amongst others, all witnessed significant SGBV incidence associated with the armed conflicts occurring in their borders (United Nations Secretary‐General, 2019). Boys are also vulnerable to sexual violence, but reliable data of prevalence is lacking (Radford & Sommarin, 2014, UNICEF, 2014). However, data collected in the DRC indicate that the number of boys (in DRC ***)*** who have experienced conflict‐related sexual violence ranges from 4% to 24% (depending upon the methodology used) (Casey et al., 2011, Duroch et al., 2011, Johnson, 2010, UNICEF, 2014).

The 2020 Covid‐19 pandemic further facilitated acts of SGBV, with some country reports of IPV increasing along with the government‐mandated lock‐downs and the stress from the loss of livelihoods and confinement (Mlambo‐Ngcuka, 2020).

##### Information and communication technology (ICT)

ICT, specifically the use of mobile phones, tablets, and web‐based communications (laptops) to address multiple issues in LMIC, has increased exponentially in the past decade (UNESCO, 2020, World Bank Group, 2016). This trend towards ICT uptake is especially true of young people with an average of 83% of those aged 18‐29 owning a mobile phone (Ippoliti & L'Engle, 2017, citing Pew Research Center, 2014). Evidence, supported by methodologically rigorous research, of the impact of using ICT in areas such as health, has indicated that *if used properly*, ICT can increase the impact of interventions and address gaps and challenges inherent with the delivery of interventions (World Bank Group, 2016). The World Bank estimates that the number of Internet users tripled from 1 billion in 2005, to 3.2 billion at the end of 2015 (World Bank Group, 2016), and that “70% of the bottom one‐fifth of the population own a mobile phone” (World Bank Group, 2018).

ICT interventions can facilitate strategies that are known to be effective for achieving certain outcomes, but face implementation challenges. For example, in the field of HIV, adherence to antiretroviral treatment is known as effective for treating HIV by reducing viral load, but lack of oversight in ensuring patients regularly adhere to their prescribed regimens is a common obstacle. The use of ICT (mobile phone reminders) has been proven as an effective strategy to ensure that patients adhere to their appointed regimens (Lester et al., 2010, Mills & Lester, 2019). The World Health Organisation recognised the need for an evidence base to support ICT use increase in health areas such as maternal, newborn and child health and HIV and AIDS, and in 2019, published a Guideline of recommendations on digital interventions, supported by a critical evaluation of evidence (World Health Organisation, 2019b). The Guideline, “identifies evidence gaps to inform member states and streamline includes future research investments” supported by contributions from eleven Cochrane reviews (Cochrane Collaboration 2019).

Stakeholders working in the area of SGBV, such as the Sexual Violence Research Initiative (SVRI) and the World Bank, have recognised and acknowledged the increased use of ICT to both prevent and respond to SGBV globally (Freeman et al., 2012, Hayes, 2014, Sexual Violence Research Initiative, 2017). As in many health areas, ICT is being used as a tool to facilitate interventions that are known to be effective in addressing SBGV (for prevention and response) outlined in globally accepted evidence‐based frameworks for preventing and addressing violence (including by not limited to SGBV) such as RESPECT (violence against women) (World Health Organisation, 2019a) and the INSPIRE (violence against children) (World Health Organisation, 2016).

Published studies on the use of ICT directly for SGBV, notably in LMIC, are scarce. Other than several recent but narrow systematic reviews that (1) provide an initial analysis and functional categorisation of mobile phone applications addressing violence against women (Eisenhut et al., 2020); (2) examine web‐ and mobile‐based delivery methods of IPV “victimization” prevention (Anderson et al., 2019); (3) examine the effect of eHealth interventions compared with standard care on reducing IPV, depression, and posttraumatic stress disorder (PTSD) among women exposed to IPV. (Linde et al., 2020); and (4) identify the effectiveness of ICT‐based IPV interventions (El Morr & Layal, 2020), a dearth of available peer‐reviewed published research exists. The preponderance of those studies that are published and readily available took place in higher income countries. There have been few attempts to identify and systematically review the research and evidence of outcomes and impact attributable to using ICT *specifically for* SGBV prevention or response in LMIC.

While evidence has been emerging examining the gender implications connected with the use of ICT including various benefits to women and girls (as well as children in general), increasing evidence has also raised concerns about the role of ICT in exacerbating SGBV (Crabtree & Geara, 2018).

#### 1.1.2 The intervention

##### What is the scope of ICT interventions for SGBV?

ICT for SGBV interventions involve a broad scope given the complex nature of SGBV in general. Further, the ICT component of an intervention, may not be an SGBV intervention per se, but its method of delivery to the end user (i.e., IPV clinical screening tools that are tablet rather than paper‐based). Modes of ICT include mobile phones, tablets and web‐based applications using laptop computers. We will include ICT interventions for the prevention of SGBV against women and children, as well as for responding to SGBV by improving survivors’ access to services, and preventing the re‐occurrence of SGBV. We will *exclude* prevention and response interventions addressing violence that is not related to socially ascribed differences between males and females. For example, we will exclude literature discussing violence connected to the disciplining of children.

The scope will also include using ICT to *achieve intermediate outcomes* that are part of causal pathways for (1) preventing SGBV against women and children in LMIC; and (2) responding to SGBV by improving survivors’ access to services. These intermediate outcomes will include those connected with evidence‐based interventions contributing to the prevention of SGBV under the RESPECT and INSPIRE frameworks.

We will *exclude* the uses of ICT if they are not specifically and purposefully used to deliver or fill gaps in prevention and response interventions for SGBV. We will exclude uses of ICT if they are not linked specifically to some aspect of gender. We will include literature that studies the unintended consequences of ICT interventions.

We will identify ICT supported interventions that have as an objective either (1) SGBV prevention; and/or SGBV response (improved access for SGBV survivors to services); or (2) an intermediate outcome that is part of the evidence‐based causal pathway to either SGBV prevention, or improved access for SGBV survivors to services (with reference to the RESPECT and INSPIRE frameworks). These intermediate outcome areas are connected with root causes of SGBV based established by global evidence. We will attempt to identify only studies related to interventions if they are delivered using ICT, and describe the role of ICT in facilitating the delivery of the intervention. These studies may compare cohorts using ICT interventions with non‐technology interventions, or with no‐intervention at all.

##### Examples

Illustrative examples of prevention interventions using ICT include: gaming applications^4^ which contain messaging aimed to sensitise and change the attitudes and behaviour of male and female adolescents to the negative consequences of gender bullying and violence and contribute; web‐based applications alerting friends and contacts to intervene if a woman feels threatened on a date; and web‐based maps warning the geographic locations where incidents of SGBV have occurred.

Illustrative examples of response interventions using ICT include: web‐based applications showing where health and counselling services for SGBV can be accessed; links to online counselling for survivors to prevent the re‐occurrence of SGBV; and WhatsApp support groups for survivors of SGBV (for a table of illustrative examples, see Appendix A).

##### Who delivers the intervention?

SGBV prevention and response interventions are typically delivered by civil society groups, non‐governmental organisations (NGOs), governments, or a collaboration of some or all that may also include universities. A separate technology partner, unless the group, NGO or government has internal technological capacity, provides the technological input, coding the ICT tool whether it is a mobile phone application and/or web‐based laptop computer application, conducting a feasibility assessment (examining the conditions such as connectivity, usability, etc.), and delivering necessary training to the targeted users of the technology. The technologists work (or are supposed to work) hand‐in‐hand with the programme specialists (e.g., child protection, gender, and SGBV specialists) to ensure the interventions supported by technology are appropriately designed deployed, and are most importantly, delivered in accordance with the “*do no harm principle*”.

SGBV interventions may also be delivered in the form of applications that can be downloaded onto phones, tablets and computers (e.g., “laptops”), and used without additional involvement of external parties.

##### Who are the targeted groups?

For the purpose of this EGM, the primary targeted groups will be:

- women and girls in LMIC;
- boys in LMIC (vulnerable to, and as survivors of SGBV); and
- others who are vulnerable to SGBV, including transgender and gender‐nonconforming persons, whether or not they identify as female.

We define “children” based upon the definition under the Convention on the Rights of the Child, to “include male and females being below the age of eighteen years unless under the law applicable to the child, majority is attained earlier” (United Nations, 1989).

However, we will also include interventions that target intermediary groups who are part of the causal pathway to preventing SGBV against women and children, and increasing access to SGBV services for survivors including preventing the recurrence of SGBV:

- potential perpetrators (i.e., men and boys) in LMIC;
- intimate partners in LMIC;
- service workers (e.g., health, social, police) in LMIC;
- organisations working in the area of SGBV or in any of the outcome areas relating to the prevention and response of SGBV identified under the RESPECT and INSPIRE frameworks; and
- communities and community leaders in LMIC.

##### Prevention/response

In addition to conceptualising interventions as intermediate or primary in terms of their targeted user, we will also consider whether the outcomes are for prevention or response using the following framework:

- ***Primary prevention***: Aimed at the whole community or at men and boys specifically to stop SGBV before it occurs. Addresses root causes of violence (White Ribbon Australia, 2016). Examples include men's engagement work and SGBV awareness initiatives in general. Community may also include institutions as well as populations, such as school systems, healthcare networks, peacekeeping forces, etc.
- ***Secondary prevention*** **:** Focuses on preventing violence from continuing or escalating. Aimed at individuals and groups at risk of being exposed, or at perpetrators of violence. May include home visits from social workers to household members who are at risk or violence; or behavioural change programmes for men with anger management problems (White Ribbon Australia, 2016). At the institutional level, secondary prevention encompasses “risky environments”, for example, schools that have incidences of SGBV amongst girls, localities where sex work is common and SGBV has been reported (especially amongst those who engage in survival sex work), forced migration response camps, etc.
- ***Tertiary prevention*** (*includes response*): Aimed at survivors and perpetrators of SGBV. Implemented after the violence has occurred and focuses on minimising the impact of violence, restoring health and safety, and preventing from occurring again (Carmody et al., 2014, White Ribbon Australia, 2016). Ideally, SBGV response interventions should include a tertiary prevention component, and we will note those response interventions which do.

#### 1.1.3 Why it is important to develop the EGM

SGBV is a persistent global phenomenon. ICT represents a potential facilitator for addressing SGBV, however, very little knowledge exists regarding its use, effectiveness, and feasibility within the context of SGBV. This scarcity of evidence frustrates important government, NGO and activist efforts to better understand ICT's potential. An EGM is a needed tool for better understanding this evidence landscape including: description of where ICT work is being done (context); focus population; whether it is to prevent or respond to SGBV; and the quality and the diversity of the body of evidence that exists.

There are few to no systematic reviews identifying evidence around the use of ICT for outcomes related to SGBV against women and children in LMIC. Those that exist are limited in scope and focus on higher income regions (Anderson et al., 2019). While systematic reviews that address the evidence connected to preventing SGBV do exist, as well as do systematic reviews that address the use of ICT to achieve other health outcomes (e.g., relating to HIV, maternal health, etc.), virtually no review that brings together ICT and SGBV exists.

Given this context, as well as the broad scope of SGBV and ICT interventions, an EGM is a critical first step in identifying clusters of evidence that can be further meta‐analysed as systematic reviews. It is necessary to understand what the evidence precisely is—and if meta‐analysis is even possible. For this reason, we felt that a traditional systematic review would be too limiting in scope to accurately capture the evidence landscape of ICT and SGBV. We anticipate that little published research exists, and an EGM is therefore necessary to systematically highlight and identify what and where these gaps are. This assists key stakeholders and activists to set research priorities. We intend to develop the evidence clusters we identify into separate, future systematic reviews if possible.

Further, as the use of ICT expands at such a rapid pace both within and outside the context of SGBV, the need for methodologically rigorous research requires advocacy amongst both the ICT and SBGV research communities. We do not anticipate many rigorous studies meeting the eligibility criteria to be identified in the review, but we believe that determining a baseline of existing evidence, as few as they may be, is an essential starting point for an advocacy strategy encouraging more rigorous studies on the topic.

To further underscore the timeliness and importance of this study, the authors take note that emerging reports show a correlation between the Covid‐19 pandemic and increasing GBV, especially IPV (Chukwueke, 2020, Mlambo‐Ngcuka, 2020). Accordingly, we will explore emerging evidence around SGBV in Covid‐19 contexts, and whether ICT has a role to play. For example, the Gender‐Based Violence Information Management System in Mali generated data demonstrating a 35% increase in SGBV between April 2019 and April 2020 (United Nations Population Fund, 2020). This SBGV trend has also been noted in other African countries.

Additionally, an EGM that locates evidence connected to the operationalisation and implementation of ICT for preventing and responding to SGBV is not only warranted, but critical for implementers to comply with the principle of “do no harm” when delivering interventions in potentially vulnerable contexts. This EGM will provide a necessary baseline of the evidence for using ICT for SGBV prevention and response interventions for women and children in lower‐ and middle‐income nations.

## 2 OBJECTIVES

*The primary objective of this EGM is to answer the following question:*

1. What is the state of the evidence connected with the of the use of information and communications technologies (ICT) for preventing and responding to sexual and gender‐based violence (SGBV) against women and children in lower‐ and middle‐income countries (LMIC)?
  a. The EGM will provide a structured and accessible contextual framework for research to stakeholders and policymakers in SGBV and ICT.
  b. The EGM will identify gaps in the available ICT and SGBV evidence.
  c. The EGM will identify clusters of evidence suitable for systematic review.
  d. The EGM will look for, and build connections between related areas of research in ICT and SGB.

*In addition to* (1), *we will also answer the following questions based upon the available evidence we find:*

2. Does the use of ICT prevent SGBV against women and children in LMIC?
3. How effective is ICT at improving access to services for SGBV survivors in LMIC?
4. Does the use of ICT contribute to effectively achieving intermediate outcomes that lead to the prevention of SGBV against women and children, and/or improving access for SGBV survivors to response services in LMIC?
5. What are the enabling factors associated with the implementation of ICT and SGBV interventions?

## 3 METHODS

### 3.1 Evidence and gap map: Definition and purpose

EGMs represent a relatively new approach to identifying and classifying available research and evidence for a broad scope or topic, such as ICT and SGBV. EGMs offer a rigorous and systematic process to “map” what evidence exists and where also gaps in evidence exist. EGMs are ideal for topics where little understanding of the research landscape exists; or those that incorporate several potential interventions with various outcomes, but may lack widely available studies. EGMs differ from systematic reviews in that they identify clusters of evidence that may be further analysed by meta‐analysis, with each cluster representing a potential systematic review in itself (Saran & White, 2018).

An EGM is ideal for SGBV and ICT precisely because the scope of SGBV and ICT encompasses several potential intervention designs with various target populations and outcomes. An EGM offers a systematic approach to locate and analyse what evidence exists for SGBV and ICT, as well as highlight evidence gaps. While EGMs do not include meta‐analysis per se—the clusters they identify are often readily developed into individual systematic reviews. In search strategy and methodological rigour, however, EGMs differ little from systematic reviews.

Systematic reviews require precision with inclusion and exclusion criteria to properly meta‐synthesise data. Given that we expect little, but diverse, available evidence for SGBV and ICT, a systematic review would likely include very little studies and miss other key evidence that is necessary to understand the broader context of SGBV/ICT research. An initial EGM, in contrast, would allow us to first identify what evidence clusters do exist, and further develop these into individual reviews if possible.

### 3.2 Framework development and scope

The EGM framework will inform the inclusion and exclusion criteria of the EGM.

The EGM framework is being developed through consultation with stakeholders (key informant interviews), a global steering committee of SGBV and ICT experts, and through reference to existing SGBV evidence‐based evidence (e.g., RESPECT and INSPIRE frameworks), theories and policies. In addition to the EGM, we are also conducting a landscape review of ICT and SGBV interventions in LMIC that will capture work being done by activists, civil society, NGOs, and other groups that are not represented or published in traditional academic and research settings (and therefore likely to not be identified by the systematic searches of peer‐reviewed literature).

### 3.3 Stakeholder engagement

We have launched a steering committee of SGBV and ICT experts, including representatives from the global South, who are assisting in the conceptualisation of the framework. The committee includes academics, civil society actors, and activists. The committee has been assisting us to identify key stakeholders for informant interviews to further help guide the EGM. Additionally, we are conducting an in‐depth landscape review to help identify where ICT and SGBV interventions are being implemented on the ground. The landscape review will identify other key stakeholders for interviews and feed into the EGM.

### 3.4 Conceptual framework

**Figure 1:**
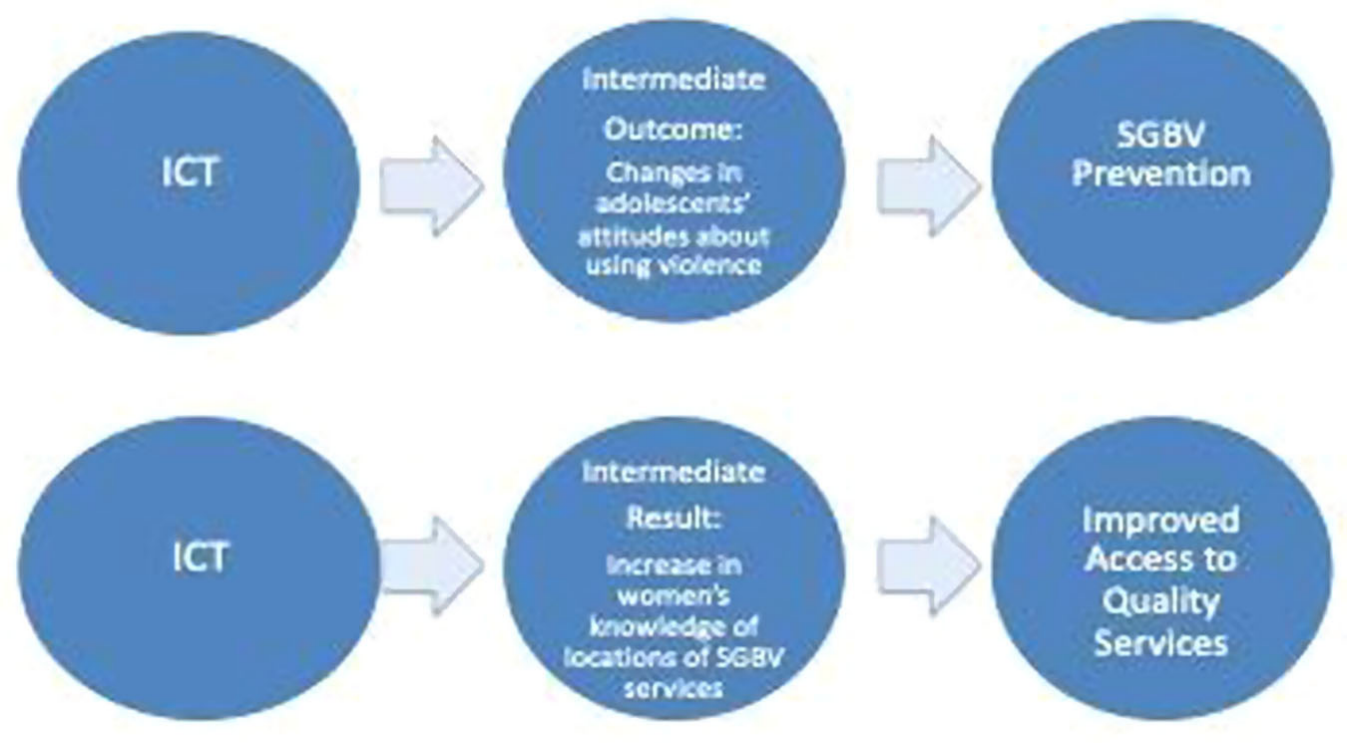
Intermediate outcomes and causation

Because of challenges measuring primary SGBV outcomes, especially prevention, different groups may be targeted as mediators (intermediaries) for achieving intermediate outcomes that contribute to, or are inferred, based upon available evidence, to contribute to, SGBV prevention and response primary outcomes. The research team acknowledges that interventions, for the purpose of ultimately preventing and/or responding to violence, may have the objective of achieving an *intermediate outcome* that contributes to the primary outcome (or impact) of either preventing SGBV or responding to SGBV (e.g., facilitating access to services, including the prevention of the SGBV recurrence for survivors of SGBV). Often there is more than one intermediate outcome, attributable to multiple interventions, which together form a causal pathway leading to the primary outcome (or impact) such as SGBV prevention (e.g., women's economic empowerment and strengthening relationship/life skills) (World Health Organisation, 2019a) (see Figure 1).

We will include studies that evaluate intermediate results that may be components of *Theories of Change* that contribute to or are prerequisites leading to primary outcomes (or impact). Often it is difficult to measure primary prevention outcomes or impact (e.g., a reduction in SGBV prevalence). Established evidence around the prevention of violence supports inferences that achieving one or more intermediate outcomes will necessarily lead to, or contribute to prevention (reduced SGBV prevalence), or effective response interventions (greater access to services). We anticipate that studies may tend to more likely evaluate the effectiveness of an intervention in achieving an intermediate outcome. Based upon evidence, we may be able to infer that the intermediate outcome will either directly lead to, or is a necessary ingredient for achieving the primary outcome/impact (e.g., SGBV prevention or improved access to SGBV services).

We will identify studies that target and measure outcomes on groups other than women and children. These targeted intermediary groups include SGBV service providers (social workers, health providers, etc.), potential and actual SGBV perpetrators (including intimate partners), community members, and organisations.

The World Health Organisation's RESPECT Women framework for the prevention of violence against women (including SGBV) is useful to conceptualise intervention SGBV outcomes (World Health Organisation, 2019a). The RESPECT framework conceptualises risk and protective factors for SGBV across four environmental levels: individual, interpersonal, community, and society. These divisions offer an important roadmap to consider SGBV outcomes that go beyond individual attitudes, knowledge, and behaviours; and look towards structural outcomes at both the community and society level. Similarly, the INSPIRE Seven Strategies for Ending Violence Against Children (World Health Organisation, 2016) presents evidence‐based strategies for preventing and addressing violence, including SGBV, against children. The RESPECT and INSPIRE frameworks will inform the EGM's outcome dimensions.

### 3.5 Dimensions

The conceptual dimensions used for coding the EGM will reflect key elements of ICT and SGBV intervention design, organised by: *type of ICT intervention, target population (or user) and intervention outcome*.

First, we will look at *target users*, or those for whom the intervention is intended. *Primary targets* are those who are vulnerable to SGBV, or survivors. This includes especially women and children. This may also include boys, men, and those who are transgender or gender non‐confirming. *Intermediary targets* are key actors who fall in the intermediate of SGBV's causal pathway. This includes potential or actual perpetrators of SGBV, first responders, organisations, and all others who may influence intermediate outcomes that contribute, or ultimately lead to SGBV primary prevention and access to services outcomes such as community health workers, or medical and social welfare professionals.

The EGM data will be organised by targeted user according to two main dimensions of ICT: *intervention* and *outcome*. The EGM will be laid out according to two axes: *x* and *y*. One axis will present types of ICT interventions. The other axis will lay out intervention outcomes and impacts. While we anticipate that many outcomes will likely measure individual and interpersonal SGBV‐related *attitudes, knowledge and behaviours*, following evidence‐based existing frameworks (i.e., RESPECT and INSPIRE), we will also consider structural outcomes at the community and societal level. We also anticipate that some evidence may relate to ICT intervention evaluation (feasibility, sustainability, etc.). We anticipate using broader outcome codes with more detailed filtering to capture additional nuance for the final EGM. We acknowledge that some overlap may exist between the dimension codes. Interventions which have more than one objective or outcome will be coded more than once as appropriate. However, we will attempt to avoid the overuse of doubling coding.

#### 3.5.1 Types of study design

- Systematic reviews of experimental or quasi‐experimental studies.
- Experimental (e.g., randomised control trials and other experiments with random assignment, including quasi‐randomised controlled trials [QRCTs] where participants are allocated by, e.g., alphabetically, without revealing personal identifiable information; non‐randomised controlled trials [NRCTs] where participants are allocated by other actions controlled by the researcher; non‐randomised studies, where allocation is not controlled by the researcher and two or more groups of participants are compared)
- Quasi‐experimental with well‐defined comparison group: that includes a well‐defined comparison group. non‐randomised controlled trials, cohort studies, case‐control studies, and cross‐sectional analytical studies.
- Rigorously designed qualitative research: guided by precise and clear research questions (RQ); with adequate data collection to address RQ; interpretation of results substantiated by data, and coherence between qualitative data sources, collection, analysis and interpretation. Descriptive qualitative evidence related to implementation or cost‐effectiveness of ICT and SGBV is particularly of relevance.

We will include studies without comparisons of cohorts if they examine elements of ICT implementation useful to SGBV and ICT stakeholders, especially those that examine cost‐effectiveness, feasibility of implementation, or acceptability of a given intervention.

#### 3.5.2 Types of intervention/problem

The use of ICT is not necessarily the intervention itself, but a means to support the underlying intervention (e.g., tool for communicating messages connected with changing social norms for SGBV prevention), or to address obstacles and challenges in the delivery of an intervention (e.g., communicating essential information to hard‐to‐reach populations regarding locations of SGBV response services). Accordingly, we will categorise interventions by type of ICT application. We will identify ICT interventions according to common types of ICT *applications*, adapting, in part, frameworks developed in the mHealth field, such as the mHealth and ICT Framework for Reproductive, Maternal Newborn and Child Health (RMNCH) (Labrique et al., 2013).

Ideally, an ICT intervention will be compared to an existing non‐ICT‐based intervention—for example, tablet‐based screening tools compared to the same tool in paper form, or web‐based workshops on gender attitudes compared to in‐person workshops.

Examples of types of ICT applications (some of which may overlap) may include:

1. Education and behaviour change communication
2. Data collection and reporting
3. Electronic decision support (information, protocols, algorithms, checklists
4. Messaging
5. Provider‐to‐provider communication
6. Provider training and education
7. Mapping (of services, incidents of violence, etc.)
8. Gaming
9. Hotlines
10. Referrals

For inclusion in our search, all interventions must have gender‐related outcomes (see Appendix A).

#### 3.5.3 Types of population (as applicable)

We will include studies that focus on two types of populations living in LMIC:

- ***Primary***, or those vulnerable or survivors of SGBV: women and children, especially girls, those vulnerable to other forms of SGBV (ex. transphobic violence).
- ***Intermediaries*** or those intermediate in the SGBV causal pathway: potential perpetrators, perpetrators, first responders, and any others intermediary agents (community health workers, social workers, organisations, etc.).

We envision two maps within the EGM—one for interventions designed for primary targets and one for intermediaries. Additional tabulation of population type within the two maps will also be included.

#### 3.5.4 Types of outcome measures (as applicable)

We will identify ICT supported interventions that have as outcome either (1) the primary outcome/impact of SGBV prevention; and/or SGBV response (improved access for SGBV survivors to services); or (2) an intermediate outcome that is part of the causal pathway to either SGBV prevention, or improved access for SGBV survivors to services.

With regard to studies with primary outcomes/impact, we do not anticipate identifying many studies that measure changes in SGBV prevalence. We are more likely to identify studies that measure changes in the uptake of SGBV‐related response services.

The intermediate outcomes may reflect any of the evidence‐based strategies for preventing SGBV reflected in the RESPECT and INSPIRE frameworks such as transformed knowledge, behaviours, beliefs and norms, environments made safe, social or economic empowerment of women, reductions in poverty, relationship skills strengthened (World Health Organisation, 2016, 2019a), or multiple intermediate outcomes.

We will search only for intermediate outcomes when there is an explicit connection with SGBV. For example, we will exclude poverty reduction interventions (a RESPECT strategy) that do not have an explicit SGBV intent connection. We will exclude interventions that create safe environments (another RESPECT strategy) without a specific intent connection to SGBV prevention.

These intermediate outcomes may be related to SGBV prevention or response. Examples include:

- Changes in knowledge and awareness by those vulnerable to SGBV about means for avoiding and preventing SGBV. Example: *a mobile phone app that helps women recognise risk factors for IPV*.
- Changes in knowledge and awareness by SGBV survivors about where and how to access SGBV services. Example: *a web‐based format that allows SGBV survivors to report SGBV incidents to police from their mobile phones and directs to local medical services for first‐response*.
- Changes in social norms, attitudes and behaviours amongst men and boys, and intimate partners regarding SGBV which ultimately should lead to reducing incidents of IPV. Example: *a gaming app that helps adolescent boys develop more gender‐equitable attitudes and behaviours*.
- Changes in community social norms, attitudes and behaviours towards SGBV. Example: *a social media campaign targeting community attitudes or beliefs regarding common SGBV myths, which ultimately should lead to a reduction of SGBV in the community*.
- Service providers building improved capacity and capabilities (knowledge) in providing SGBV services to SGBV survivors. Example: *phone‐based tools to assist social workers in better identifying SGBV risk factors or signs of SGBV when making home visits, which ultimately should lead to a reduction of SGBV, increased access to services and/or the re‐occurrence of SGBV*.
- Changes in the economic empowerment of women and family household incomes and improved relationship skills between intimate partners. Example: *a mobile phone application that guides women in women‐led village savings and loan associations, and provides relationship building curriculum for discussion which should contribute to the reduction of IPV*.

#### 3.5.5 Other eligibility criteria

We are focusing on interventions implemented in LMIC, but may refer to interventions implemented in higher income nations to provide context and for comparison in the descriptive narrative and analysis.

##### Types of settings

We will include ICT interventions that have home‐based individual end‐user settings (mobile phone, tablet, or other web‐based settings); as well as those that may encompass clinical settings (ex. ICT‐based SGBV screening tools). We will include interventions in LMIC. Interventions in higher‐income settings will be excluded from the EGM, but we may refer to interventions in higher‐income settings to provide context in the descriptive narrative and analysis.

The following settings and contexts will be included, but not necessarily limited to:

1. Humanitarian and emergency settings (including refugees and internally displaced persons or “IDPs”)
2. IPV
3. Children in the armed forces
4. Trafficking
5. Schools
6. Conflicts, war, fragile states
7. Mobile children and women (including migrants)

### 3.6 Search methods and sources

#### 3.6.1 Electronic searches

Relevant studies will be identified through electronic searches of bibliographic databases, research networks, government policy databanks and internet search engines. The searches will include studies published from 2005 and forward (The search dates are restricted as the results of the use of ICT for health interventions were rare, if not existent, prior to 2005). Results in English, Portuguese and Spanish (languages spoken by the reviewers) will be reviewed. The bibliographies of relevant reviews and included studies will be used to identify additional references for review. We will conduct forward citation searching using the website Google Scholar as this database produces similar results to other search engines.

Any changes in eligibility criteria will be agreed prospectively between the members of the review team. These will be documented and reported as a discrepancy from protocol in the manuscript. In the advent of a change in eligibility, we will re‐screen citations.

Search terms will be developed based on terminology representative of implementation and dissemination research and include search filters used in previous reviews. We will particularly leverage globally accepted terminology around SGBV in globally accepted frameworks and guidance including INSPIRE: Seven Strategies for Ending Violence Against Children (World Health Organisation, 2016), RESPECT Women: Prevention Violence Against Women (World Health Organisation, 2019a), Essential Services Package for Women and Girls Subject to Violence, Sexual Violence Researcher Initiative, Together for Girls, and the Violence Against Women Working Group of the International Federation of Obstetrician‐Gynecologists (FIGO). Regarding ICT related terminology, we will draw from body of studies addressing the use of “mHealth” and “eHealth” that have become standardised in the use of digital technology. The search strategy for PubMed is presented under “Sample Search Terms” below and will be adapted for the other databases by an experienced librarian, who also will conduct the searches.

#### 3.6.2 Grey literature

We will review relevant grey literature, developing a separate search strategy including all of the following elements:

1. *Searches of grey literature databases* such as ProQuest Dissertations and Theses; TROVE; Open Grey etc.
2. *Targeted Google searches* that will be based on the search terms used with electronic databases.
3. The *targeted screening of relevant sector and implementation websites (landscape review)*, including content from civil society, feminist groups NGOs and other activist web‐based content relevant to the prevention and response of SGBV in LMIC.

As SGBV and ICT span multiple disciplines and contexts, social science, public health, education, humanitarian, and ICT related electronic databases will be searched. Search terms will vary by database but will generally include three blocks of terms and appropriate Boolean or proximity operators, if allowed: blocks will include terms that address (1) intervention; (2) population; (3) outcomes.

*Bibliographic databases to be searched:*

- Association for Computing Machinery (ACM) Digital Library
- African Journals Online (AJOL)
- Business Source Complete
- Campbell Collaboration Library searched through July 2019
- Centre for Reviews and Dissemination Databases
- CiteSeerX
- ClinicalTrial.gov
- Cochrane Library
- EBSCO
- Education Resources Information Center (ERIC, via ProQuest)
- EPPI‐Centre Systematic Reviews
- Google Scholar
- Ideas/Economist online
- IMISCOE
- International Bibliography of the Social Sciences
- Journal of Social Work Practice
- LILACS (Latin American and Caribbean Health Sciences Literature)
- MEDLINE
- Mendeley
- PILOTS (Published International Literature on Traumatic Stress)
- ProQuest dissertations & theses A&I
- PsychARTICLES
- PsycINFO
- PubMed
- ScienceOpen
- Sexual Violence Research Initiative (SVRI)
- Social Care Online
- Social Science Citation Abstract
- Social Science Research Network (SSRN)
- SocIndex
- SpringerLink

#### 3.6.3 Searching other resources

Copies of relevant documents from Internet‐based sources will be made. We will record the exact URL and date of access.

##### Snowballing

Reference lists of included studies and relevant reviews will be searched for potential new literature.

##### Personal contacts

Personal contacts with national and international researchers will be considered in order to identify unpublished reports and on‐going studies.

#### 3.6.4 Sample search terms (not exhaustive)

(ICT [tiab] OR Information*‐ communication*‐ technolog* [tiab] OR tablet_ OR mobile‐phone [tiab] OR web [tiab] OR cell‐phone [tiab] OR electronic [tiab] OR digital [tiab] OR gam* [tiab]) OR podcast (tiab)) AND (“Sexual gender based violence” [tiab] OR “gender‐based violence” [tiab] OR rape [tiab] OR SGBV [tiab] OR GBV [tiab] OR intimate‐partner‐violence [tiab] OR IPV [tiab] OR FGM OR female‐genital‐mutilation [tiab] OR violence‐against‐children [tiab] OR sexual‐harassment [tiab] OR domestic‐violence [tiab] OR child‐abuse [tiab] OR IPV [tiab] OR physical‐violence [tiab] OR sexual*‐abuse [tiab] OR traffick* [tiab] OR transphobia [tiab] OR homophobia [tiab] OR women [tiab] OR child* [tiab] OR gender [tiab] AND econ* [tiab] OR poverty [tiab] OR empower* [tiab] OR norm* [tiab] OR social [tiab] OR relation* [tiab].

### 3.7 Analysis and presentation

#### 3.7.1 Report structure

The report will follow Campbell's guidelines for structure for EGM: an executive summary, background, methods, results and conclusion.

Tables and figures related to types of SGBV ICT interventions identified, along with relevant information to their population of interest, location, and outcome measures will be included in the final report.

#### 3.7.2 Filters for presentation

Additional dimensions may be important to stakeholders such as (1) country or region, (2) age group, (e.g., 0–2, 3–5, 5–11), (3) delivery channel/place (e.g., home, school, health facility, community). In the hard copy multiple 2 × 2 representations of the EGM may be reported. In the online interactive map, the additional dimensions can be used as a filter to select studies meeting those criteria. Provide detailed definitions for each additional dimension. Describe how the different dimensions will be captured in the map.

Given the broad scope of ICT and SGBV, it will be useful to include additional filters that highlight studies by the following (in addition to the dimensions described above):

- Region and country
- ICT delivery (e.g., mobile phone application, tablet, web‐based/Internet)
- SGBV context (e.g., IPV, trafficking, extreme poverty, humanitarian, conflict/post‐conflict, IDP/refugee, etc.)
- Specific target population (e.g., women, girls, adolescent boys, men, first responders, communities, organisation)
- Vulnerable population
- Sector(s) implicated: health, justice/legal, social welfare, education, multi‐sector, etc.
- Type of study
- Size of cohort

#### 3.7.3 Dependency

Define the unit of analysis for primary studies. Define whether each item represents a report or a study, and what will be done when there are multiple reports for a single study, or when a report covered multiple studies.

We will include individual studies with multiple reports as a single study. This includes any studies that are included in more than one systematic review.

### 3.8 Data collection and analysis

#### 3.8.1 Screening and study selection

Two independent reviewers will first independently screen studies’ titles and abstracts. Disagreements between reviewers will be resolved by consensus. Potentially eligible studies will then be retrieved in full text and these full texts will be reviewed for eligibility, again using two independent reviewers. Disagreements between reviewers will again be resolved via discussion and consensus. If we cannot determine eligibility due to missing information in a report, we will contact the study authors for this information. The completed review will include a table of studies excluded at the full text level of screening and provide rationale for each exclusion decision. We will also publish a PRISMA flow chart to report the screening process and results (PRISMA‐P Group et al., 2015).

#### 3.8.2 Data extraction and management

Two review authors, also unblinded to author or journal information, will independently extract information from the included trials. This information will be recorded in a data extraction form that will be piloted before initiation of the review. Discrepancies between reviewers regarding data extraction will be resolved by consensus or if required via a third reviewer. One reviewer will transcribe information from data extraction forms into Rev Man meta‐analysis software and included study tables. Data transcription will be checked by a second reviewer. Machine learning wil not used in this EGM.

In addition to the standard categories used in coding of studies such as year of study, setting and other contextual features, target population(s), study method(s), sample sizes outcomes, etc., we will include categories regarding classification of ICT (e.g., mobile phone, tablet, web‐based computer, etc.), general characteristics of, including age categories of ICT users, connectivity and other ICT enabling conditions (e.g., literacy), type of intervention: (1) Prevention: primary, secondary, tertiary) or 2) Response (technical sector: e.g., health, justice, psycho‐social, education, etc.). We will draw from commonly and generally accepted frameworks for analysing SGBV for children (INSPIRE), and for women (e.g., Essential Services Package for Women and Girls Subject to Violence); Minimum Standards for Prevention and Response to Gender‐based Violence in Emergencies.

See Appendix B for a copy of the coding sheet with illustrative categories.

#### 3.8.3 Tools for assessing risk of bias/study quality of included reviews

##### Quality of evidence assessment of reviews

While we do not anticipate identifying many reviews, we will use the AMSTAR 2 tool (Shea et al., 2017) to assess the quality of reviews and assess risk of bias.

##### Quality of evidence assessment of studies

The overall quality of evidence for each study, we will be rated using the GRADE system (Guyatt et al., 2011) by two reviewers, with any disagreements resolved via consensus, or if required by a third reviewer. The GRADE system defines the quality of the body of evidence for each review outcome, describing the extent to which one can be confident in the review findings. The GRADE system requires an assessment of methodological quality, directness of evidence, heterogeneity, and precision of effect estimates and risk of publication bias. GRADE quality ratings (from “very low” to “high”) will be used to describe the quality of the body of evidence for each review outcome. All assessments of the quality of the body of evidence (e.g., downgrading or upgrading) will be justified and documented. For assessing qualitative evidence, we will utilise the GRADE‐ CERqual tool.

##### Risk of bias

Risk of bias will be assessed independently by two reviewers using the risk of bias tool described in the *Cochrane Handbook for Systematic Reviews of Interventions* (Higgins et al., 2019). Judgments made will be justified with information included in studies or related documents. If these documents are not publicly available, this will be explicitly stated. If required, a third reviewer will adjudicate discrepancies regarding the risk of bias that cannot be resolved via consensus.

## CONTRIBUTIONS OF AUTHORS

- Content: William C. Philbrick, Jacob R. Milnor, Madhu Deshmukh, Patricia N. Mechael.
- EGM methods: Jacob R. Milnor, William C. Philbrick.
- Statistical analysis: William C. Philbrick, Jacob R. Milnor.
- Information retrieval: William C. Philbrick, Jacob R. Milnor.

## DECLARATIONS OF INTEREST

The authors declare that there are no conflict of interests.

## SOURCES OF SUPPORT

### External sources

- The project to develop the EGM is funded by a non‐for‐profit organisation with no competing interest. The funder is not involved in editorial decisions or decisions related to publishing, USA.

## Footnotes

1 SGBV, sometimes referred to as gender‐based violence (GBV) is “any harmful act of sexual, physical, psychological, mental, and emotional abuse that is perpetrated against a person's will and that is based on socially ascribed (i.e., gender) differences between males and females” United Nations Office for the Coordination of Humanitarian Affairs (United Nations Office for the Coordination of Humanitarian Affairs, 2019).

2 Monitoring and Evaluation to Assess and Use Results. MEASURE Evaluation. MEASURE Evaluation is a cooperative agreement awarded by the U.S. Agency for International Development (USAID) to the Carolina Population Centre at the University of North Carolina at Chapel Hill and five partner organisations: ICF International, John Snow Inc., Management Sciences for Health, Palladium, and Tulane University (MEASURE Evaluation, 2020) MEASURE Evaluation works closely with USAID, its country missions and counterparts to improve collection, analysis and presentation of data to promote better use of data in planning, policy‐making, managing, monitoring, and evaluating population, health, and nutrition programmes, and such, maintains the SGBV subarea within its Family and Reproductive Health Indicators Database.

3 Global and regional estimates of violence against women: prevalence and health effects of IPV and non‐partner sexual violence are difficult to estimate with preciseness (Breiding et al., 2015). Physical, mental, and sexual effects linked with IPV include adolescent pregnancy, unintended pregnancy in general, miscarriage, stillbirth, intrauterine haemorrhage, nutritional deficiency, abdominal pain and other gastrointestinal problems, neurological disorders, chronic pain, disability, anxiety and posttraumatic stress disorder (PTSD), as well as noncommunicable diseases such as hypertension, cancer and cardiovascular diseases (World Health Organisation, 2013).

4 The term “app” can be used interchangeably with “application”.

## APPENDIX A TABLE OF ILLUSTRATIVE INTERVENTIONS

**Table:**

Intervention objective (intermediate result or primary outcome):	ICT Intervention	Targeted group(s) (primary or intermediate)	Outcome/Impact
Increase in knowledge of physical locations of high SGBV incidents to avoid (Primary).	Mobile SMS and web‐based mapping application alerting of high SGBV risk locations	Women (primary)	Secondary Prevention: reduction in incidence of SGBV in certain locations
Mobile phone or web‐based referrals to SGBV service providers (Intermediate)	Mobile phone referrals applications	Service providers (intermediate)	Improve access to services; including prevention services: increase in number or proportion of SGBV survivors who access SGBV service providers
Provide children access to SGBV counselling services (Primary)	Childline/Child Helpline mobile phone counselling	Children (primary)	Improve access to services and tertiary prevention: increase in number of children who access services; decrease in number/proportion of SGBV child survivors who experience a re‐occurrence of SGBV
Prevent further violence to children (Intermediate)			
Change social norms around violence between partners (intermediate)	Mobile phone messaging around masculinity and violence	Intimate Partners (intermediate)	Primary prevention and Secondary prevention: Reduction of number or rate of SGBV incidents
Changing social norms around SBGV against children (prevention)	Gaming applications on mobile phones and tablets	Children and adolescents, women (primary and intermediate)	Primary, Secondary and Tertiary prevention; improve access to services: reduction of number or rate of SGBV incidents and/or reoccurrence
Educating about how to best respond to SBGV (response)			

## APPENDIX B CODING SHEET EXAMPLES

**Table:**

1: Study characteristics	Definition	Example
1.1: Organization	1 = University, 2 = Government Entity, 3 = Contract Research Firm, 4 = NGO, 5 = IGO, 6 = Civil Society, 7 = Mixed, 8 = Other (specify)	(4) CARE
1.2: Publication Language	1 = English, 2 = Portuguese, 3 = Spanish	
1.3: Methods Used	1 = Quantitative, 2 = Qualitative, 3 = Mixed‐methods, 4 = other (specify)	
1.4: Participatory Design	1 = Yes, 2 = No	
1.5: Theoretical Framework Used	1 = Yes (specify), 2 = No	(1) RESPECT Framework
1.6: Study Duration	1 = One Day or Less, 2 = One Week or Less, 3 = One Month or Less, 4 = One Marking Period or Less, 5 = One Semester or Less, 6 = One Year or Less, 7 = More Than One Year	
1.7: Control Group	1 = Yes, 2 = No	

2: Geographics	Definition	Example
2.1: Region	1 = Africa, 2 = Asia, 3 = Central America, 4 = Europe, 5 = North America, 6 = South America, 7 = Southeast Asia	
2.2: Country Income	1 = Lower Income Country, 2 = Middle Income Country, 3 = High Income Country	
2.3: Country (Name)	(specify)	South Africa

3: Population characteristics	Definition	Example
3. 1: Target Population	1 = Women Only (18+), 2 = Girls Only (<18), 3 = Women and Girls, 4 = Men (18 + ), 5 = Boys Only (<18), 6 = Boys and Men, 7 = Children (Boys and Girls <18), 8 = LGBTQ, 9 = First Responders, 10 = Other (specify)	(2) Girls Age 12–17
3.2: Key populations	1 = Yes (specify) 2 = No.	(1) HIV positive populations
3.3: Environment	1 = Urban, 2 = Rural, 3 = Peri‐urban, 4 = Mixed, 5 = Not Specified	
3.4: Setting/Context	1 = Extreme Poverty, 2 = Natural Emergency/Disaster, 3 = Armed forces, 4 = Conflict/post‐conflict, 5 = trafficking, 6 = Migration, 7 = IDP/Refugee, 8 = School, 9 = Sex work, 10 = Medical, 11 = Social Services, 12 = Other (specify)	(8) Children in Secondary Schools in Sao Paulo, Brazil.
3.5: Sectors Implicated	1 = Health, 2 = Justice/legal, 3 = social welfare, 4 = Education, 5 = Other, 6 = Coordination across sectors	(1) Frontline community healthcare workers in Sub‐Saharan Africa

4: ICT/Intervention Design	Definition	Example
4.1: SGBV Prevention or Response	1 = Prevention and/or 2 = Response	(2) Linkage to first response services
4.2: ICT Medium	1 = Mobile Phone, 2 = Tablet, 3 = Web‐Based Laptop or PC, 4 = Podcast, 5 = Other (specify)	(1) Mobile Phone application
4.3: Intervention Type	1 = Games/Gaming, 2 = Education/Awareness, 3 = Referrals, 4 = Reporting, 5 = Case Management, 6 = Training, 9 = Access to Services, 10 = Community Mobilization, 11 = Data Collection and Management, 12 = Counseling,13 = Diagnostics; 14 = Messaging; 15 = Decision Making Protocols; 16 = Mapping; 17 = Entertainment	(5) Mobile Phone App to Facilitate Case Management for IPV Survivors
4.5: Intervention Sustainability Addressed	1 = Yes, 2 = No	(2) ICT Implementation Plan Does Not Include Provision for future financing

5: Outcomes	Definition	Example
5.1: Outcome Target	1 = Individual 2 = Interpersonal, 3 = Community, 4 = Societal	(4) Increased IPV Reporting
5.2: Outcome Scope	1 = Relationship Skills Strengthened, 2 = Empowerment of Women (or target group); 3 = Services Access Ensured; 4 = Poverty Reduction; 5 = Safe Environments; 6 = Prevention of Child and Adolescent Abuse; 7 = Improved SGBV Attitudes, Beliefs, Norms; 8 = Parent or Caregiver Support; 9 = Implementation and Enforcement of Laws, 10 = Diagnostics, 11 = Response Services Strengthened	(5) School Teachers Trained on Recognizing SGBV Risk in Adolescent Girls at Secondary Schools, and taking appropriate actions
5.2: Impact Measured	Y = Yes, N = No 1 = Level of SGBV; 2 = Referrals; 3 = Access to Services	Y(1) National SGBV Incidence
5.3: Includes Prevention of SGBV Recurrence	1 = Yes, 2 = No, 3 = N/A	(1) First Response with Linkage to Follow Up Social Services and judicial protection
